# CF_3_SO_2_X (X = Na, Cl) as reagents for trifluoromethylation, trifluoromethylsulfenyl-, -sulfinyl- and -sulfonylation and chlorination. Part 2: Use of CF_3_SO_2_Cl

**DOI:** 10.3762/bjoc.13.273

**Published:** 2017-12-19

**Authors:** Hélène Chachignon, Hélène Guyon, Dominique Cahard

**Affiliations:** 1 UMR 6014 CNRS COBRA, Normandie Université, 1 rue Tesnière, 76821 Mont Saint Aignan, France

**Keywords:** chlorination, fluorine, sulfur, trifluoromethylation, trifluoromethylsulfenylation, trifluoromethylsufinylation, trifluoromethylsulfonylation

## Abstract

The recent progresses of the application of trifluoromethanesulfonyl chloride, CF_3_SO_2_Cl, in the formation of C–CF_3_, C–SCF_3_, C–SOCF_3_, and C–Cl bonds are summarised in this second part of a two-part review published back-to-back on both sodium trifluoromethanesulfinate, CF_3_SO_2_Na, (Part 1) and trifluoromethanesulfonyl chloride, CF_3_SO_2_Cl (Part 2). There are many reactions in common between these two reagents but it should be noted that CF_3_SO_2_Cl reacts under reductive conditions while CF_3_SO_2_Na requires oxidative conditions. Electrophilic chlorination is obviously the exclusive preserve of CF_3_SO_2_Cl that has been exploited with emphasis in enantioselective chlorination.

## Introduction

In the preceding paper, we described the various uses of sodium trifluoromethanesulfinate in direct trifluoromethylation, trifluoromethylsulfenylation, trifluoromethylsufinylation and trifluoromethylsulfonylation reactions. We now focused this second part of the review on the similarly diverse uses of trifluoromethanesulfonyl chloride plus chlorination. This review appears in two parts that are published back-to-back. We encourage the readers to refer to Part 1 for a general introduction in the field [1].

## Review

Trifluoromethanesulfonyl chloride (alternate name: triflyl chloride), CAS No. 421-83-0, MW 168.53, is a colourless liquid (bp 29–32 °C) soluble in dichloromethane, tetrahydrofuran and dioxane [2]. Up to recently, the predominant use of CF_3_SO_2_Cl was for triflate and triflamide formation. Indeed, CF_3_SO_2_Cl reacts with oxygen nucleophiles to generate triflate derivatives as highly electron-withdrawing substituent in order to act in nucleophilic substitutions and metal-catalysed coupling reactions as an excellent leaving group [2]. The reaction of CF_3_SO_2_Cl with nitrogen nucleophiles provides trifluoromethanesulfonamide (triflamide) derivatives, which are used in drugs and agrochemicals [3]. The *C*-trifluoromethylsulfonylation is less reported than the corresponding *O*- and *N*-trifluoromethylsulfonylations, although the resulting triflone group is an important synthetic tool for further functionalisation [4–5]. These sulfonylation reactions will not be further detailed hereafter. Instead, CF_3_SO_2_Cl, which is experiencing an advanced level of growth for the installation of the CF_3_ moiety onto a wide range of substrates, alone or simultaneously with the chlorine atom or the sulfonyl group, is the focus of this review. The direct introduction of CF_3_S and CF_3_S(O) motifs also occupies a prime position in this review.

### 1 Trifluoromethylation

#### C_sp3_–CF_3_ bond-forming reactions

**Scheme 1:**
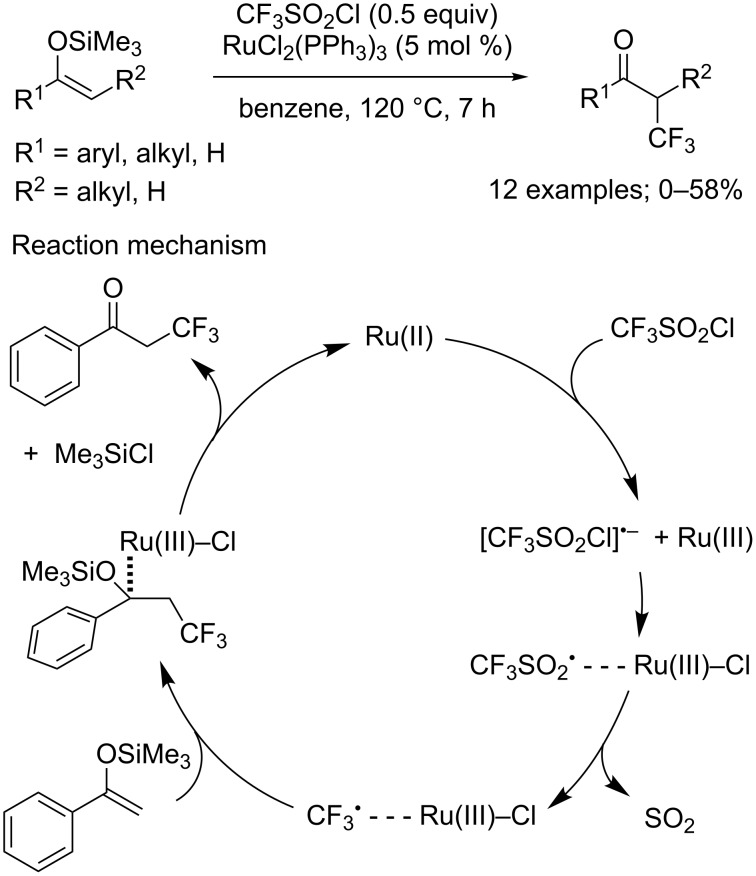
Trifluoromethylation of silyl enol ethers.

**Trifluoromethylation of silyl enol ethers and enol acetates:** After their original reports on the trifluoromethylation of aromatics in 1990 (C_sp2_–CF_3_ bond-forming reactions; see later in the text, Scheme 24) [6–7], Kamigata and co-workers studied silyl enol ethers in 1997 in trifluoromethylation reactions. Kamigata’s group reported that in the presence of RuCl_2_(PPh_3_)_3_, in benzene at 120 °C, silyl enol ethers could furnish the corresponding α-trifluoromethylated carbonyls in low to moderate yields (Scheme 1) [8]. Nonetheless, important competition between the introduction of the CF_3_ group or a Cl atom was invariably observed in various ratio depending on the nature of the substrates. As for the mechanism of the reaction, the authors proposed a radical pathway that involved Ru(II)/Ru(III) metallic species (Scheme 1).

**Scheme 2:**
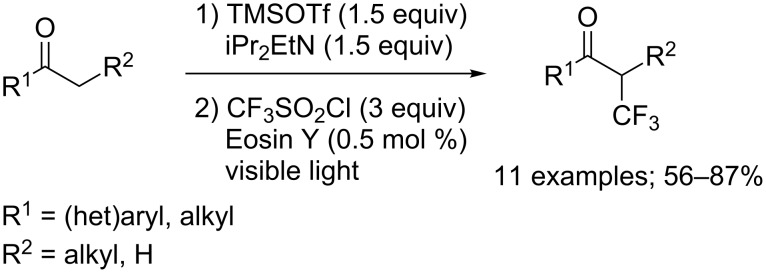
Continuous flow trifluoromethylation of ketones under photoredox catalysis.

The trifluoromethylation of silyl enol ethers can also be adressed in a continuous-flow procedure. To do so, the appropriate ketones were transformed in situ into the corresponding silyl enol ethers, which were then reacted with CF_3_SO_2_Cl in the presence of Eosin Y under visible light irradiation (Scheme 2) [9]. Acetophenone derivatives with various substitution patterns as well as aliphatic or heteroaromatic ketones were equally well tolerated. This methodology offered the advantage of minimising the chlorination side reaction, consequently resulting in higher yields indifferently of the substrate.

**Scheme 3:**
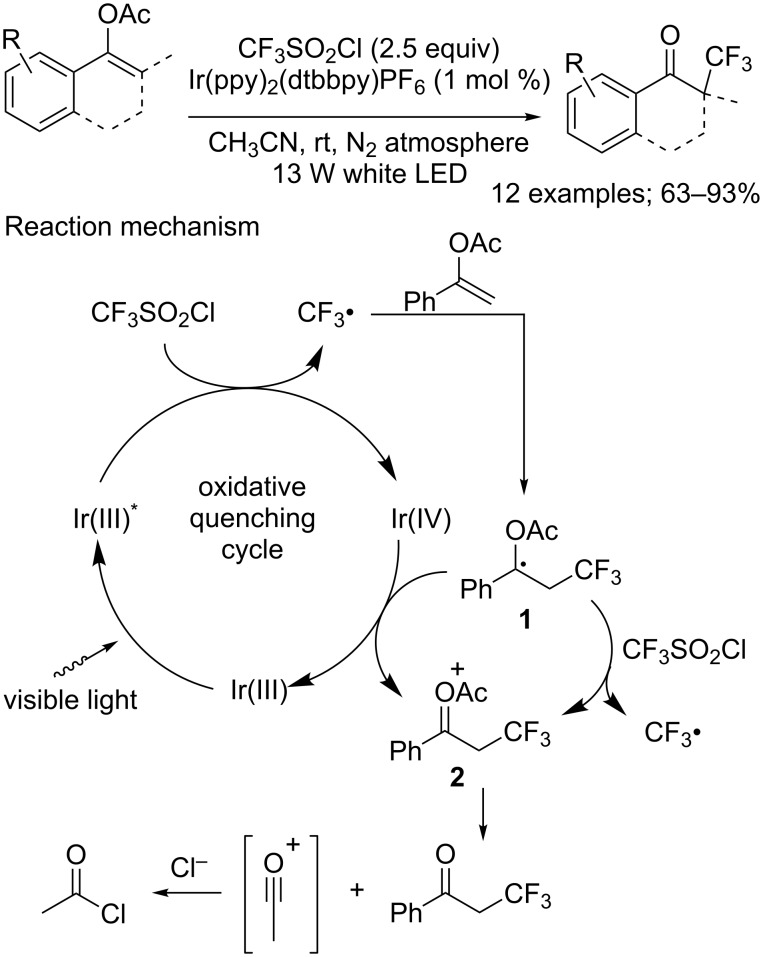
Trifluoromethylation of enol acetates.

Enol acetates as another type of masked enol(ates) also proved to be appropriate substrates to access α-trifluoromethylated ketones (Scheme 3) [10]. In the presence of 1 mol % of (4,4'-di-*tert*-butyl-2,2'-bipyridine)bis[(2-pyridinyl)phenyl]iridium(III) hexafluorophosphate, Ir(ppy)_2_(dtbbpy)PF_6_, various aryl enol acetates carrying electron-donating or electron-withdrawing groups were converted into the corresponding products in high yields. Moreover, the reaction was compatible with cyclic and acyclic branched enol acetates. Quite interestingly, when the reaction was performed using an aryl or alkylsulfonyl chloride, instead of trifluoromethanesulfonyl chloride, no extrusion of the SO_2_ moiety was observed, and the sulfonated products were recovered. The reaction mechanism involved excitation of the iridium catalyst under visible light to generate an Ir(III)* species, which was then oxidatively quenched by CF_3_SO_2_Cl to furnish Ir(IV) and the CF_3_ radical. Said radical was added on the substrate to form the radical species **1**, which yielded the cationic intermediate **2** through oxidation by Ir(IV). The oxidation of compound **1** by means of CF_3_SO_2_Cl, regenerating the trifluoromethyl radical in the process, was also considered. Intermediate **2** was ultimately converted into the final product after liberating an acetyl cation, which was captured by a chloride anion to give acetyl chloride (Scheme 3).

**Scheme 4:**
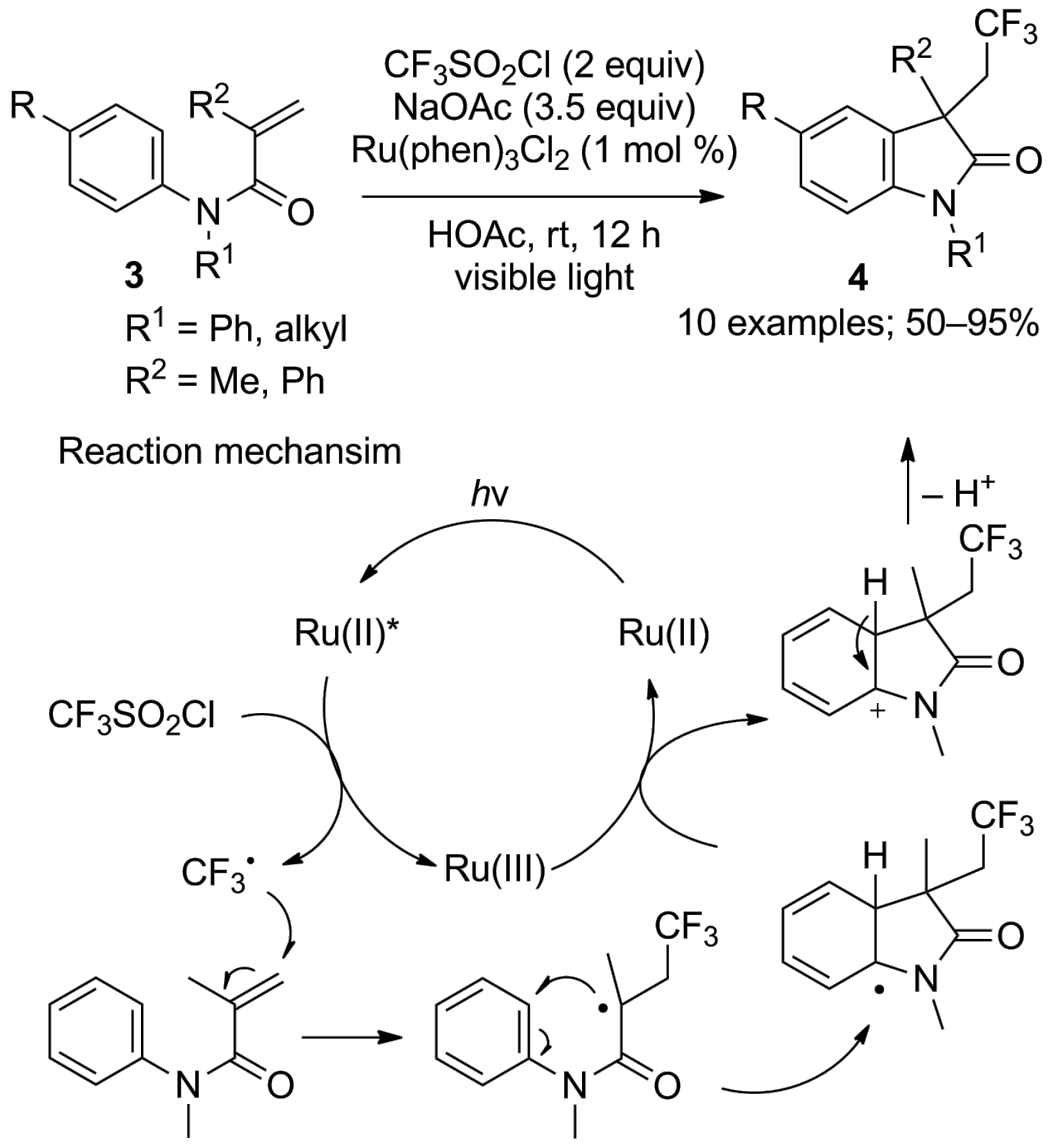
Photoredox-catalysed tandem trifluoromethylation/cyclisation of *N*-arylacrylamides: a route to trifluoromethylated oxindole derivatives.

**Trifluoromethylation of olefins with cascade reactions:** The most widely described type of reactions in which CF_3_SO_2_Cl and molecules carrying a C=C double bond are involved are actually cascade reactions that include a cyclisation or a group migration step. In this context, the acrylamide motif was a notably popular object of research, and served in several tandem trifluoromethylation/cyclisation processes. Dolbier and co-workers first proposed the use of *N*-arylacrylamides **3** to access trifluoromethylated 3,3-disubstituted 2-oxindoles **4** under photocatalytic conditions (Scheme 4) [11]. In the presence of Ru(phen)_3_Cl_2_ (phen = phenanthroline), a variety of *N*-arylacrylamides *para*-substituted on their aryl moiety by electron-donating or electron-withdrawing groups were converted into the corresponding oxindoles with similarly good yields. However, the reaction was compatible only with acrylamides bearing methyl or phenyl as R^1^ and R^2^ groups; for example, *N*-acyl and *N*-sulfonyl amides failed to react.

**Scheme 5:**
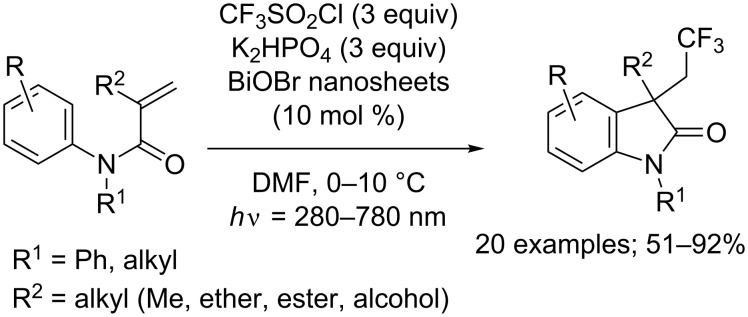
Tandem trifluoromethylation/cyclisation of *N*-arylacrylamides using BiOBr nanosheets catalysis.

Interestingly, Zhang and co-workers demonstrated that this reaction could be performed as well using bismuth oxybromide (BiOBr) nanosheets instead of a ruthenium complex as the photocatalyst (Scheme 5) [12]. The reaction unfortunately suffered from the same limitations. However, the scope of application was extended to substrates carrying more diverse R^2^ groups such as ethers, esters or alcohols.

**Scheme 6:**
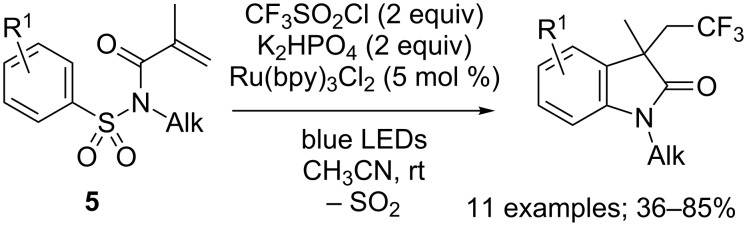
Photoredox-catalysed trifluoromethylation/desulfonylation/cyclisation of *N*-tosyl acrylamides (bpy: 2,2’-bypyridine).

In 2015, Yang, Xia and co-workers reported that trifluoromethylated oxindole derivatives could also be accessed from *N*-tosylacrylamides **5**, via a similar pathway including an additional desulfonylation step (Scheme 6) [13]. Both electron-withdrawing and electron-donating groups on *para*-position of the aryl ring were tolerated, and provided comparable yields. On the other hand, the presence of a substituent in *meta*-position led to the formation of two regioisomers. As for *ortho*-substituted substrates, they furnished even more complex reaction mixtures, probably because of steric hindrance.

**Scheme 7:**
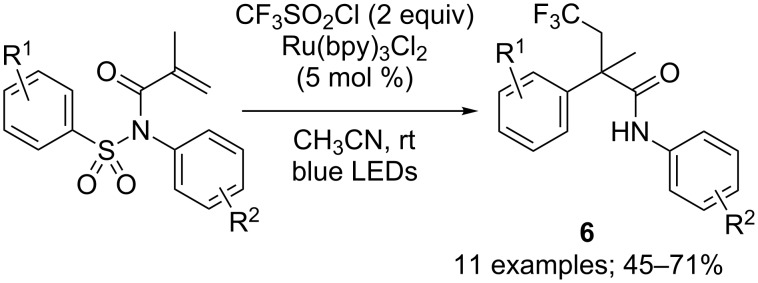
Photoredox-catalysed trifluoromethylation/aryl migration/desulfonylation of *N*-aryl-*N-*tosylacrylamides.

The most influential parameter however proved to be the nature of the substituent linked to the nitrogen atom. Indeed, when replacing the alkyl group by an aryl moiety, a totally different product was obtained predominantly: the α-aryl-β-trifluoromethyl amide **6**. This compound was determined to be issued from a trifluoromethylation/1,4-aryl shift/desulfonylation cascade reaction (Scheme 7).

This reaction could be performed on various *N*-aryl,*N*-tosylacrylamides with moderate to good yields. The nature of the substituents of the sulfonamide group showed little influence on the efficiency of the process. On the contrary, better results were obtained when realising the reaction on substrates featuring electron-donating groups on the phenyl ring directly bound to the nitrogen atom. It was also shown that the reaction proceeded equally smoothly when using BiOBr nanosheets catalysis [14].

**Scheme 8:**
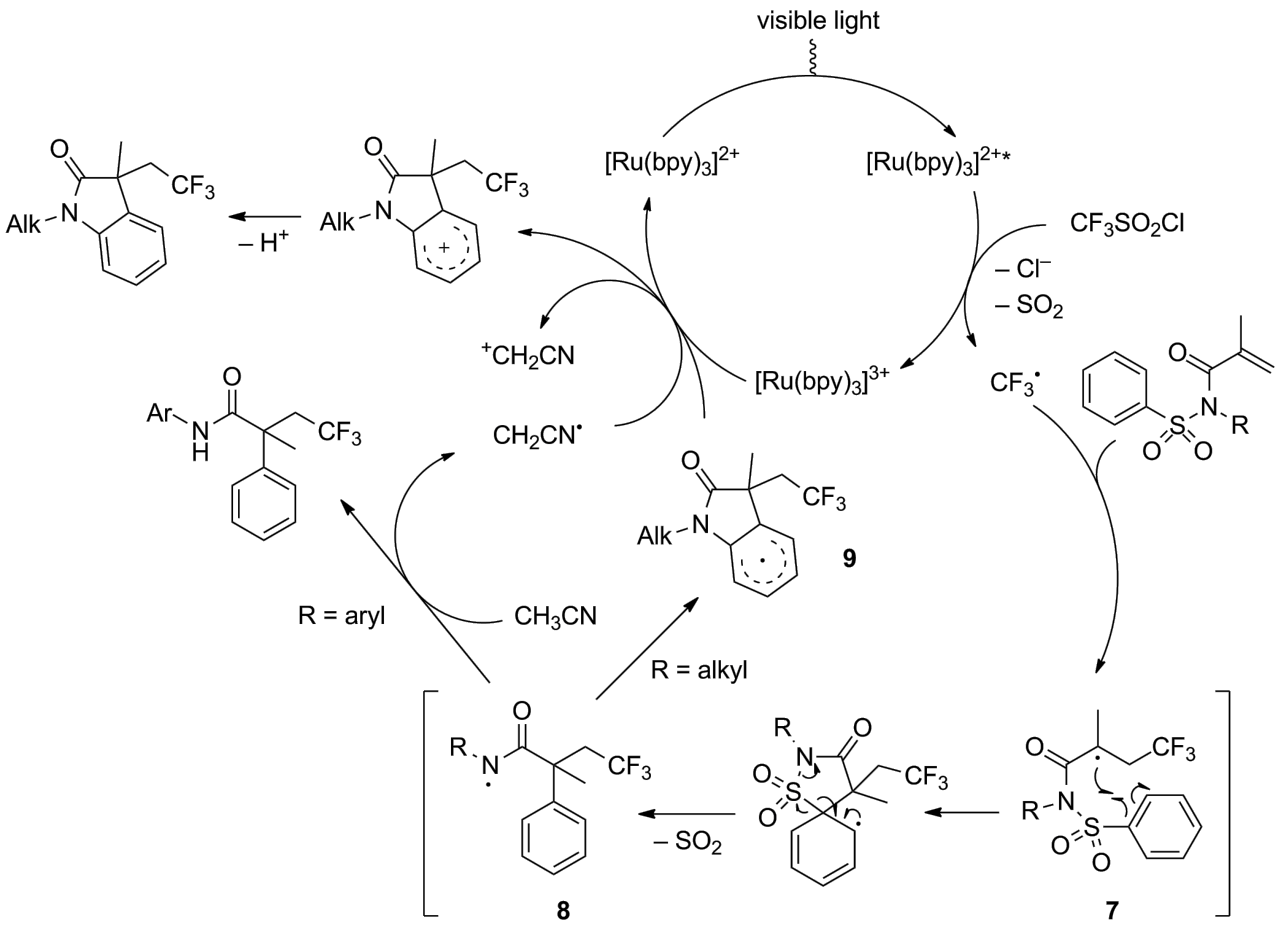
Proposed mechanism for the trifluoromethylation/aryl migration/desulfonylation (/cyclisation) of *N*-tosylacrylamides.

The proposed mechanism of these two reactions is represented in Scheme 8. Alongside the classical pathway, the trifluoromethyl radical was generated and added onto the *N*-tosylacrylamide. The obtained radical species **7** then underwent an aryl migration/desulfonylation cascade reaction to furnish intermediate **8**. In the case of an aryl substituted substrate, this nitrogen radical being stabilised, it directly performed an hydrogen abstraction on acetonitrile to lead to the corresponding α-aryl-β-trifluoromethyl amide. On the other hand, for *N*-alkylacrylamides, intermediate **8** preferentially cyclised to yield intermediate **9**, which ultimately gave access to the oxindole derivative product.

**Scheme 9:**
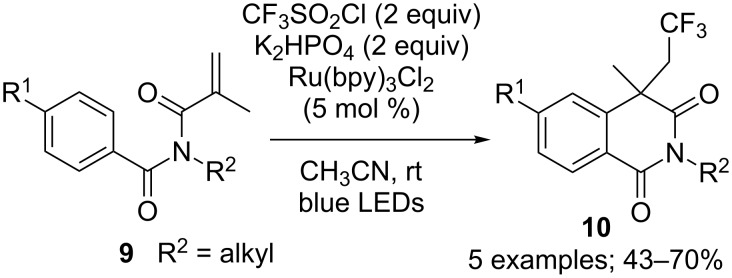
Photoredox-catalysed trifluoromethylation/cyclisation of *N*-methacryloyl-*N*-methylbenzamide derivatives.

Yang, Xia and co-workers were also interested in structurally close substrates that are *N*-methacryloyl-*N*-methylbenzamide derivatives **9**. It was found out that such compounds could take part in similar catalytic cycles, without CO extrusion, to yield trifluoromethylated isoquinolindione derivatives **10** in moderate to good yields (Scheme 9).

**Scheme 10:**
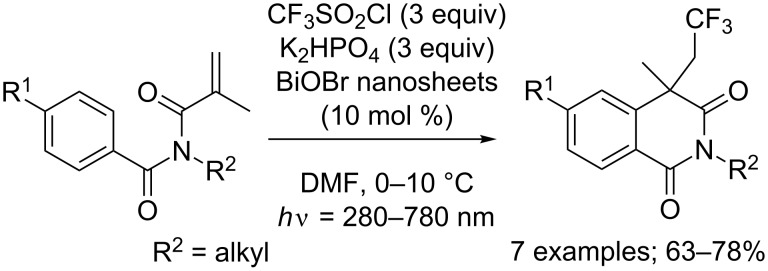
Photoredox-catalysed trifluoromethylation/cyclisation of *N*-methylacryloyl-*N*-methylbenzamide derivatives using BiOBr nanosheets.

Additionally, Zhang and co-workers reported once again that BiOBr nanosheet catalysis was also suitable to carry out this reaction [12]. The same tendencies in term of reactivity of the substrates depending on their substitution pattern was observed, and similar yields were achieved (Scheme 10). Similarly, Ir(ppy)_2_(dtbbpy)PF_6_ was also described as an appropriate catalyst for this cascade reaction [15].

**Scheme 11:**
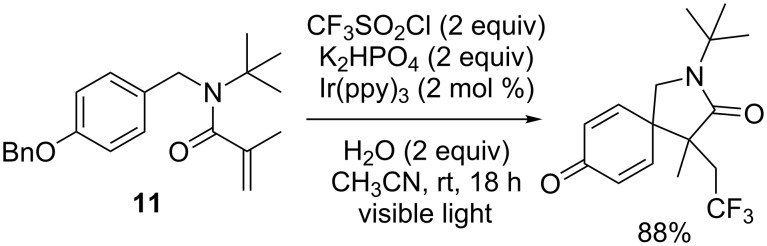
Photoredox-catalysed trifluoromethylation/dearomatising spirocyclisation of a *N*-benzylacrylamide derivative (ppy = 2-phenylpyridyl).

Another cyclisation pattern was observed for other benzylacrylamide derivatives: indeed, in the case of the *N*-benzylmethacrylamide derivative **11**, the formation of an azaspiro[4,5]decyl system through a dearomitising spirocyclisation was observed (Scheme 11) [16].

**Scheme 12:**
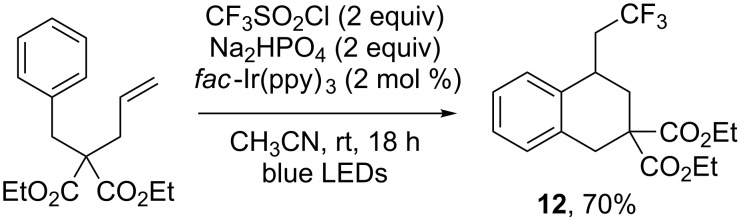
Photoredox-catalysed trifluoromethylation/cyclisation of an unactivated alkene.

Other motifs than acrylamides were also investigated for the realisation of cascade reactions including a trifluoromethylation step. This was notably the case of unactivated alkenes: Dolbier and co-workers showed that such compounds could be involved in photoredox-catalysed trifluoromethylation reactions, followed by a 6-*exo* radical cyclisation, to yield the tetralin derivative **12** (Scheme 12) [17].

**Scheme 13:**
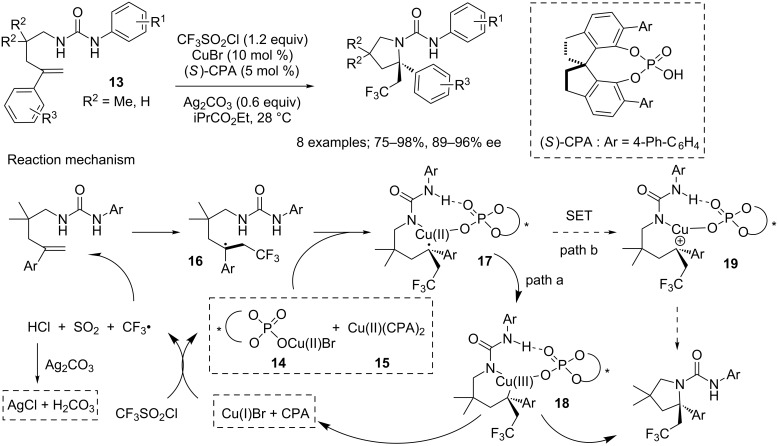
Asymmetric radical aminotrifluoromethylation of *N*-alkenylurea derivatives using a dual CuBr/chiral phosphoric acid catalytic system.

In 2017, Liu and co-workers focused on *N*-alkenylurea derivatives **13**, and from which they developed an asymmetric radical aminotrifluoromethylation methodology, based on a copper salt/chiral phosphoric acid dual-catalytic system [18–19]. This way, they could access a variety of α-tertiary pyrrolidines carrying a β-trifluoromethyl group, in high yields and enantioselectivities (Scheme 13). The reaction conditions were compatible with various substituents on both aryl rings, as well as with unbranched substrates. Interestingly, for some substrates, this method permitted to reach better results than with a previously developed approach using Togni’s reagent. The authors proposed the mechanism represented on Scheme 13. First, the trifluoromethyl radical and the chiral mono or diphosphonate Cu(II) **14** or **15** were generated via a single-electron transfer (SET) between CF_3_SO_2_Cl and the association CuBr/chiral phosphoric acid. In the process, SO_2_ and HCl were released, but the latter was scavenged by Ag_2_CO_3_, minimising its impact on the reaction process by notably avoiding hydroamination side reactions. The trifluoromethyl radical was then added onto the substrate, furnishing radical intermediate **16**, which was trapped by compounds **14** or **15** to form the radical copper species **17**. From this intermediate, two plausible pathways were considered. First, the alkyl radical can be trapped by copper phosphate to provide the copper(III) species **18**. During this step, facial selectivity originated partly from hydrogen-bonding interactions between the chiral phosphate and the N–H bond adjacent to the aryl group. Ion pairing interaction in a concerted transition state probably intervened in this phenomenon as well. The final product was then obtained after reductive elimination of species **18**. The other envisaged pathway was the oxidation of intermediate **17** through a SET to form the cationic species **19**, which would then afford the final product after a C–N bond formation.

**Scheme 14:**
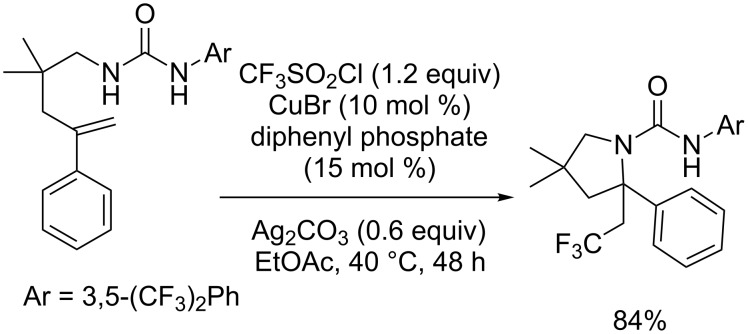
Aminotrifluoromethylation of an *N*-alkenylurea derivative using a dual CuBr/phosphoric acid catalytic system.

Liu and co-workers also proposed a racemic version of this reaction, replacing the chiral phosphoric acid with diphenyl phosphate (Scheme 14) [20].

**Scheme 15:**
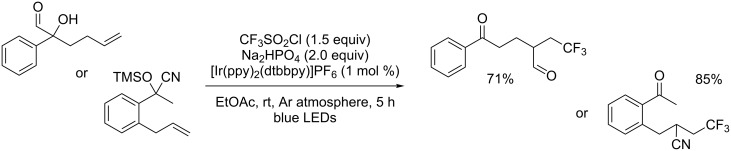
1,2-Formyl- and 1,2-cyanotrifluoromethylation of alkenes under photoredox catalysis.

Liu’s research group was interested as well in 1,2-difunctionalisation of unactivated alkenes. In this context, they developed two distinct approaches allowing to perform radical-mediated 1,2-formyl- [21] or 1,2-cyanotrifluoromethylations [22] of alkenes under photoredox catalysis. These reactions proceeded through a formyl or a cyano group migration triggered by the addition of the trifluoromethyl radical onto the alkene moiety. Both methodologies were developed using Togni’s hypervalent iodine reagent as the CF_3_ source, but it was found that they also proceeded smoothly with CF_3_SO_2_Cl (Scheme 15).

**Scheme 16:**
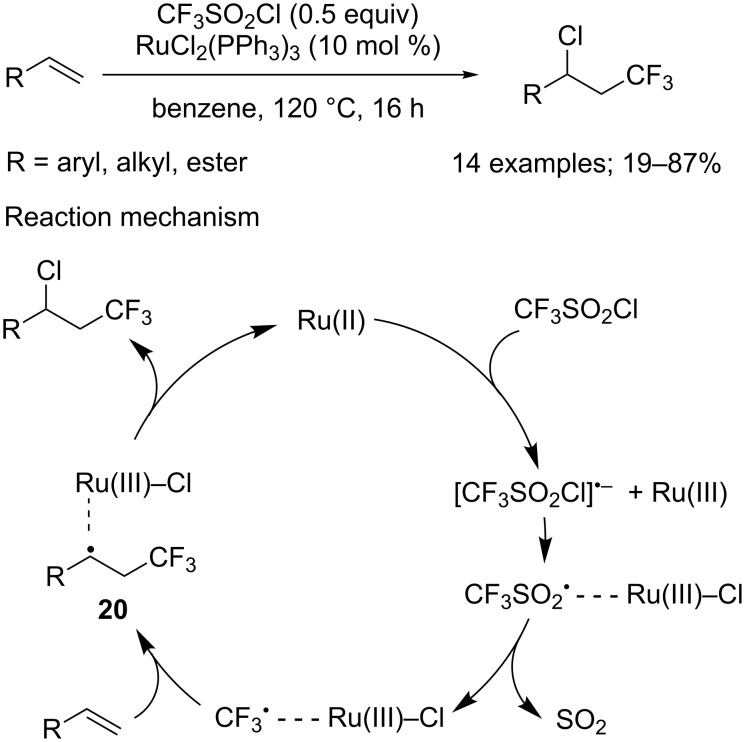
First simultaneous introduction of the CF_3_ moiety and a Cl atom onto alkenes.

**Chlorotrifluoromethylation of alkenes:** As clearly demonstrated in the works described above, CF_3_SO_2_Cl is a reliable CF_3_ source under photoredox catalysis. However, its use under similar conditions can also allow the simultaneous introduction of the CF_3_ moiety and a Cl atom onto alkenes or alkynes. Kamigata’s group was the first to report such type of transformation in 1989 [23–24]. In the presence of RuCl_2_(PPh_3_)_2_ at 120 °C, a variety of styrene derivatives as well as cyclic and acyclic alkenes were converted into their chlorotrifluoromethylated analogues (Scheme 16). Generally, the reaction proceeded particularly well with terminal and internal alkenes carrying an electron-withdrawing group. On the contrary, styrene derivatives bearing an electron-donating group provided less satisfying yields. Such results can be explained by the partial consumption of the expected product in a side dehydrochlorination reaction. Likewise, the dehydrochlorinated product was recovered exclusively when performing the reaction on 1-phenyl-1,3-butadiene, which tended to indicate that such process was all the more favoured as the conjugation of the final product increased. Cyclic olefins and dienes proved to be more problematic substrates because they raised stereoselectivity issues and afforded poor yields. As for the mechanism, the reaction followed a similar pathway as the one proposed by the same group for the trifluoromethylation of silyl enol ethers (see Scheme 1); except that radical **20** underwent a chlorine atom abstraction to furnish the chlorotrifluoromethylated product (Scheme 16).

**Scheme 17:**
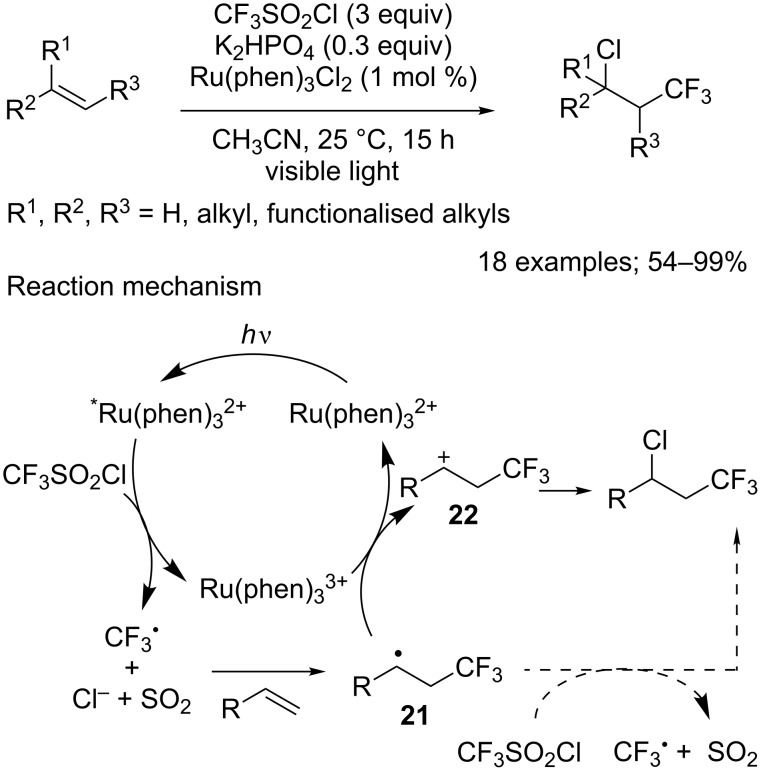
Chlorotrifluoromethylaltion of terminal, 1,1- and 1,2-substituted alkenes.

Several years later this transformation of alkenes was re-investigated under photoredox catalysis by Jung, Han and co-workers [25]. By replacing RuCl_2_(PPh_3_)_2_ with Ru(phen)_3_Cl_2_ (phen: phenanthroline) at room temperature and adding a base, a variety of alkenes furnished the corresponding chlorotrifluoromethylated products under much milder conditions and with higher yields (Scheme 17). Moreover, tuning of the reaction conditions allowed to broaden the scope of the reaction: Indeed, it was extended to terminal alkenes carrying various functional groups, such as protected amines, unprotected alcohols and aldehydes, as well as ether, ester, or amide moieties. Branched and internal alkenes also proved to be compatible with these conditions. Interestingly, no dehydrochlorination reaction was mentioned for any of the studied substrates. The reaction plausibly proceeded through a mechanism similar to the one previously proposed by Kamigata and co-workers. The SET generated CF_3_ radical attacked preferentially the less hindered carbon of the alkene to provide the more stable tertiary radical **21**. Said radical was then oxidised by Ru(phen)_3_^3+^, yielding the cationic species **22**, which was subsequently trapped by a chloride anion to afford the expected product (Scheme 17). A chain propagation pathway involving the reduction of CF_3_SO_2_Cl by radical intermediate **21** was also considered. This, however, was declared unlikely as it was observed that the reaction needed continuous irradiation to proceed efficiently.

**Scheme 18:**
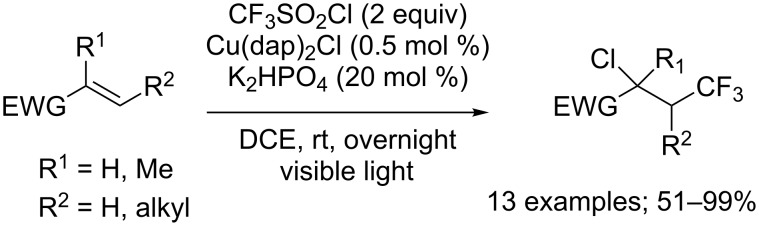
Chorotrifluoromethylation of electron-deficient alkenes (DCE = dichloroethane).

Unfortunately, Dolbier and co-workers demonstrated in 2015 that this catalytic system was inefficient when switching the substrates to electron-deficient alkenes [26]. Such compounds could nonetheless be converted into the corresponding chlorotrifluoromethylated products by replacing the Ru(II) catalyst by Cu(dap)_2_Cl (dap = 2,9-bis(*p*-anisyl)-1,10-phenanthroline) (Scheme 18). This change of reactivity can supposedly be attributed to the high reduction potential of Cu(dap)_2_Cl in the excited state, and its important ability to mediate the transfer of the Cl atom. Consequently, a variety of electron-deficient alkenes, such as *N*-arylacrylamides, acrylonitrile, acrylate and enone derivatives furnished their chlorotrifluoromethylated analogues in moderate to excellent yields. Interestingly, performing the reaction on 1,1-disubstituted alkenes did not impact the yield significantly, while 1,2-disubstituted alkenes provided slightly less satisfying results.

**Scheme 19:**
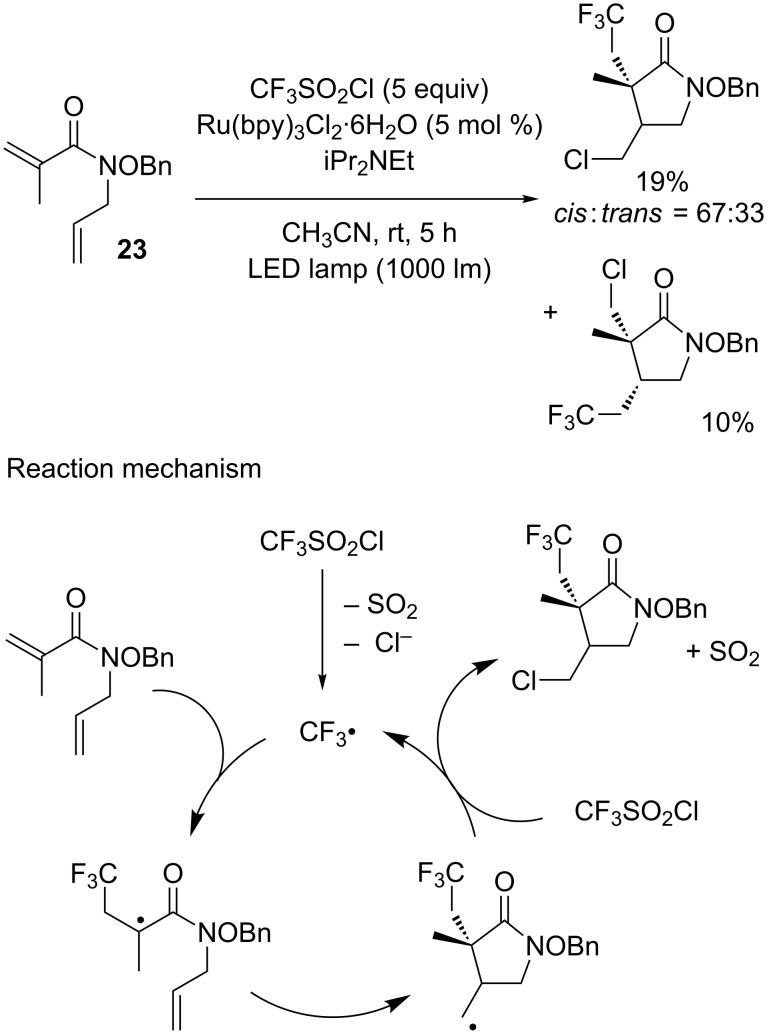
Cascade trifluoromethylation/cyclisation/chlorination of *N-*allyl-*N*-(benzyloxy)methacrylamide.

Chlorotrifluoromethylation reactions can also be included in cascade radical addition/cyclisation processes, as demonstrated by Miyabe and co-workers [27]. Thus, in typical photoredox catalysis conditions, *N-*allyl-*N*-(benzyloxy)methacrylamide **23** could undergo the addition of the CF_3_ radical, followed by a cyclisation step and a final chlorine abstraction to yield the corresponding cyclic compound, albeit in low yield and with poor regio- and diastereoselectivity (Scheme 19). Interestingly, the authors proposed a mechanism involving a chain propagation pathway, in contrast to the work of Jung and Han.

**Scheme 20:**
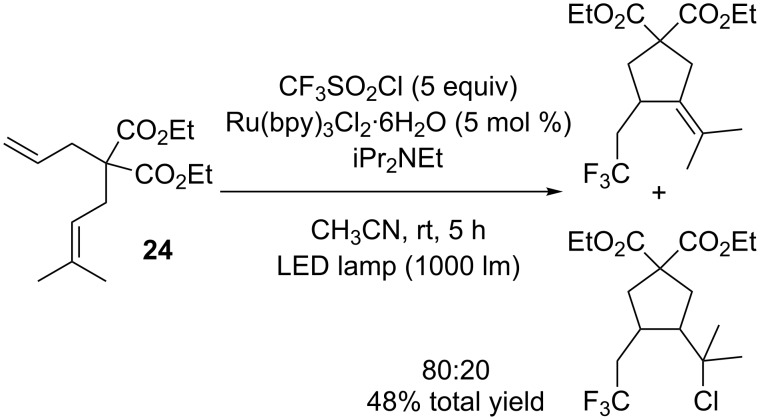
Cascade trifluoromethylation/cyclisation (/chlorination) of diethyl 2-allyl-2-(3-methylbut-2-en-1-yl)malonate.

A similar cascade reaction was also performed on diethyl 2-allyl-2-(3-methylbut-2-en-1-yl)malonate (**24**) to yield a mixture of two cyclic CF_3_ products, the main one being deprived of the chlorine atom (Scheme 20). Unfortunately, no more investigation was carried out on this type of cascade reactions.

**Scheme 21:**
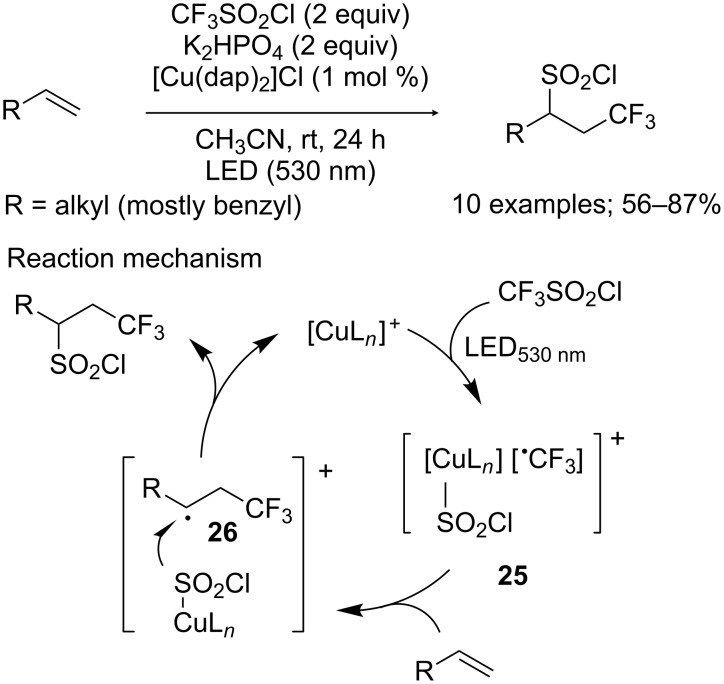
Trifluoromethylchlorosulfonylation of allylbenzene derivatives and aliphatic alkenes.

**Trifluoromethylchlorosulfonylation of alkenes:** It was previously evocated that the system CF_3_SO_2_Cl/[Cu(dap)_2_]Cl could be used for the simultaneous introduction of the CF_3_ moiety and a chlorine atom onto electron-deficient alkenes (see Scheme 18). However, Reiser and co-workers observed that such reagent combination could also be exploited for the trifluoromethylchlorosulfonylation of a limited range of alkenes [28]. Thus, allylbenzene derivatives carrying diverse substituents were successfully converted into the expected products with moderate to high yields, as well as cyclic or acyclic aliphatic alkenes (Scheme 21). Nevertheless, internal alkenes suffered from regio- and stereoselectivity issues, and often produced mixtures of isomers. On the other hand, when substrates featuring a donor atom close to the C=C double bond were submitted to these reaction conditions, chlorotrifluoromethylation was predominantly observed, which was consistent with Dolbier’s work. Moreover, similar results were obtained for styrene derivatives, although with a subsequent dehydrochlorination step. If the nature of the substrate undoubtedly played an important role in the reaction process, it was also the case of the catalyst. Indeed, the use of other usual photocatalysts such as [Ru(bpy)_3_]Cl_2_, [Ir(ppy)_2_(dtbbpy)]PF_6_ or Eosin Y favoured the introduction of the CF_3_ group and a chlorine atom, with SO_2_ extrusion. This phenomenon can be explained by the presumed ability of copper to coordinate SO_2_Cl^−^ (intermediate **25**), preventing it from decomposing into SO_2_ and Cl^−^ and consequently allowing it to be transferred as a whole onto radical **26**. However, this bonding interaction appeared to be weak enough to possibly be destabilised in the presence of a donor atom on the alkene substrate, thus favouring SO_2_ extrusion and chlorotrifluoromethylation.

**Scheme 22:**
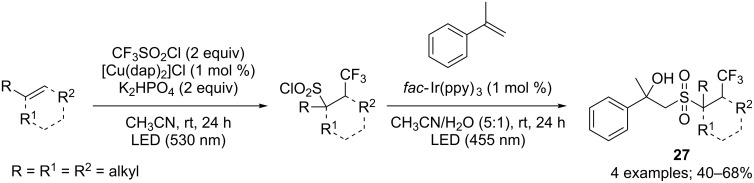
Access to β-hydroxysulfones from CF_3_-containing sulfonyl chlorides through a photocatalytic sequence.

The obtained CF_3_-containing sulfonyl chloride derivatives could then be involved in another photocatalytic sequence in the presence of α-methylstyrene and water to access β-hydroxysulfones **27** in moderate to good yields (Scheme 22) [29]. Interestingly, this process can be realised in one-pot.

**Scheme 23:**
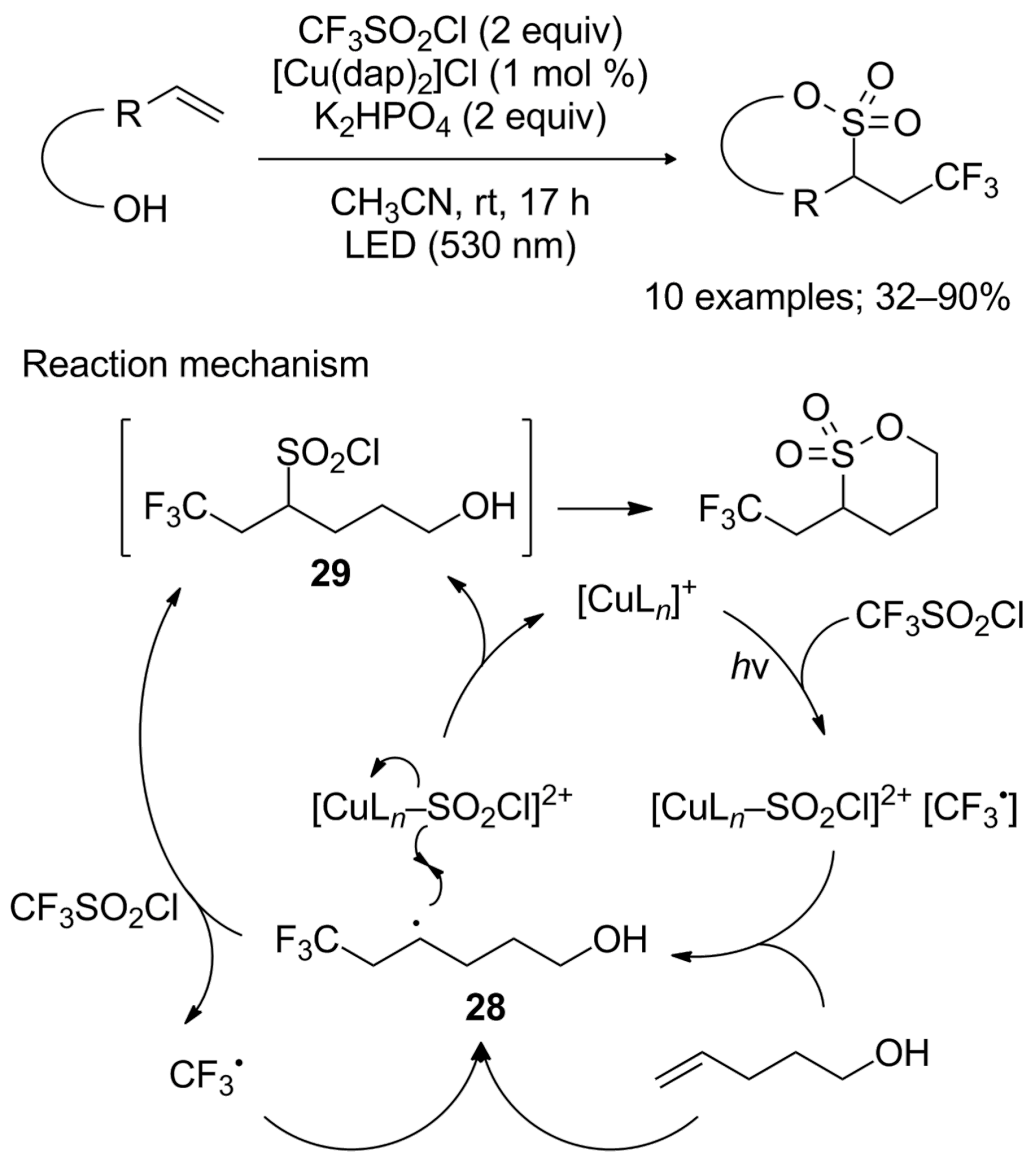
Cascade trifluoromethylchlorosulfonylation/cyclisation reaction of alkenols: a route to trifluoromethylated sultones.

Reiser and co-workers also envisioned that using alkenols as substrates in their previously developed reaction conditions could open an access to trifluoromethylated sultones via a cascade trifluoromethylchlorosulfonylation/cyclisation process [30]. The reaction indeed proceeded smoothly, furnishing γ- and δ-sultones in good to excellent yields (Scheme 23). ε-Sultones, on the other hand, proved to be more difficult to obtain. The first step appeared to be the trickiest one, as it could be anticipated considering Reiser’s previous reports. Once again, the results were strongly substrate-dependant; indeed the reaction was particularly sensitive to steric effects. The use of alkenols bearing substituents on the double bond or close to it favoured the formation of the chlorotrifluoromethylated products. The mechanism of the reaction was supposedly identical to the one proposed for the trifluoromethylchlorosulfonylation of simple alkenes, although obviously including an additional step of intramolecular cyclisation. An alternative radical chain process was also considered this time, involving notably the reaction of radical **28** with CF_3_SO_2_Cl to produce CF_3_^•^ and intermediate **29**.

#### C_sp2_–CF_3_ bond-forming reactions

**Scheme 24:**
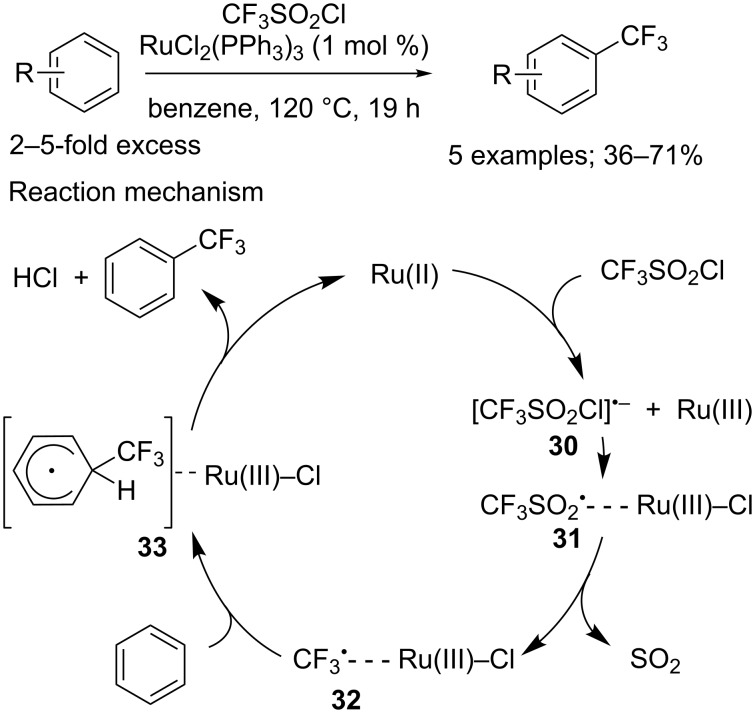
First direct C–H trifluoromethylation of arenes and proposed mechanism.

**Trifluoromethylation of arenes and heteroarenes:** The pioneering example of such transformation was reported in 1990 by Kamigata and co-workers, and described the introduction of the CF_3_ moiety onto arenes in the presence of a catalytic amount of RuCl_2_(PPh_3_)_3_ (Scheme 24) [6–7]. This reaction, however, suffered from limitations, such as its poor regioselectivity in the case of monosubstituted arenes, and its incompatibility with aromatics bearing strong electron-withdrawing groups. The authors proposed the mechanism represented in Scheme 24. A redox-transfer reaction occurred between CF_3_SO_2_Cl and the Ru(II) catalyst producing radical anion **30**, which then furnished radical **31** through homolytic cleavage. After a step of SO_2_ extrusion, the obtained trifluoromethyl radical **32** was added to the aromatic substrate to afford cyclohexadienyl radical **33**, which was converted into the expected product after a proton abstraction mediated by the R(III)–Cl species.

**Scheme 25:**
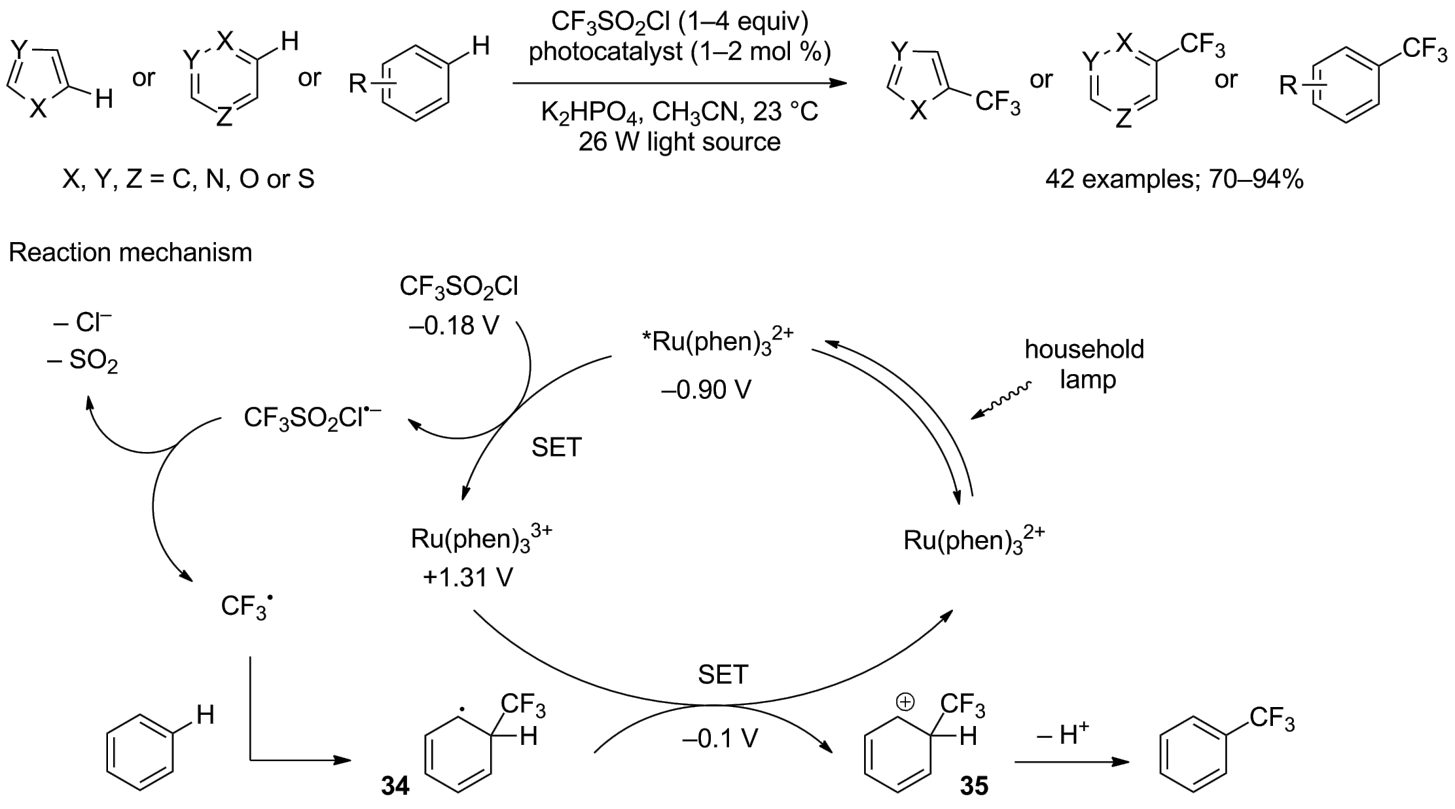
Direct C–H trifluoromethylation of five- and six-membered (hetero)arenes under photoredox catalysis.

The introduction of the CF_3_ motif on unactivated arenes was studied in more detail by MacMillan’s group in 2011 [31]. They proposed a new methodology based on the use of photoredox catalysts such as Ru(phen)_3_Cl_2_ or Ir(Fppy)_3_, (Fppy = 2-(2,4-difluorophenyl)pyridine), under the irradiation of a simple household light bulb. This way, they were able to considerably extend the scope of application of the reaction. Indeed, electron-rich five-atom heteroarenes, electron-deficient six-atom heteroarenes as well as unactivated arenes were easily converted into their trifluoromethylated analogues in high yields (Scheme 25). Although the regioselectivity of the reaction was overall excellent for heteroarenes, it proved to be less satisfying for substituted arenes. Further investigations allowed the authors to propose a detailed mechanism, represented in Scheme 25. After excitation of the ruthenium catalyst through visible light irradiation, a first SET reduction of CF_3_SO_2_Cl occurred, ultimately leading to the formation of the stabilised trifluoromethyl radical after releasing SO_2_ and chloride anion. This electron deficient radical was then added on the most electron-rich position of the arene substrate to yield cyclohexadienyl radical **34**, which was readily converted into the cationic species **35** through a second SET regenerating the Ru(II) catalyst. Finally, the cationic intermediate **35** underwent a simple re-aromatising deprotonation to yield the expected product.

**Scheme 26:**
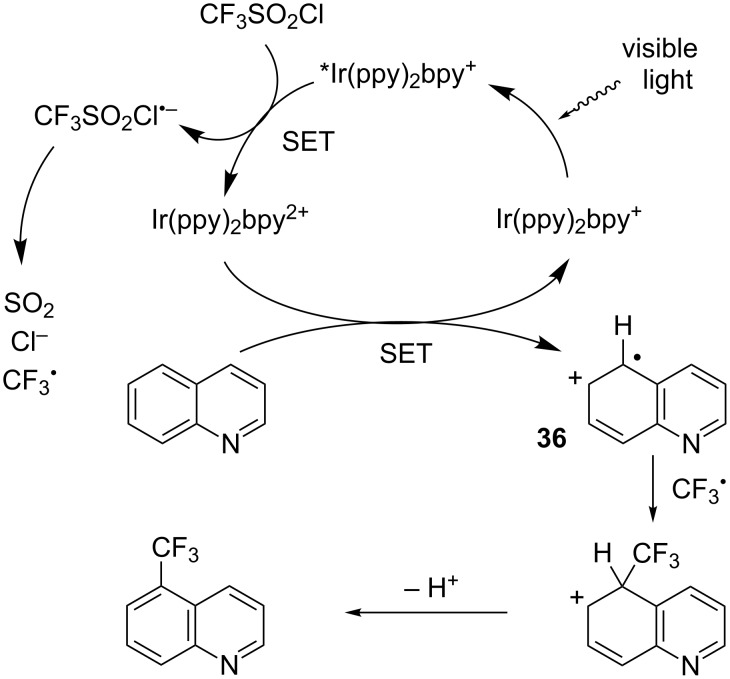
Alternative pathway for the C–H trifluoromethylation of (hetero)arenes under photoredox catalysis.

Later Wolf and co-workers conducted electrochemical investigations on this type of reaction and proposed a slightly revised mechanism in which the second SET step involved directly the substrate instead of a trifluoromethylated radical such as species **34**. The CF_3_ radical then coupled with the generated radical intermediate **36** (Scheme 26) [32].

**Scheme 27:**
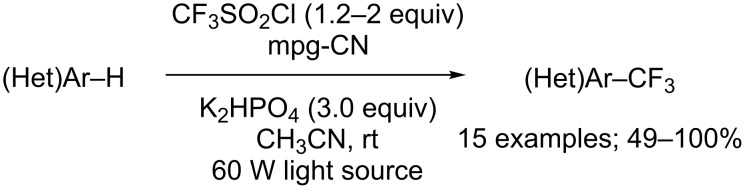
Direct C–H trifluoromethylation of five- and six-membered ring (hetero)arenes using heterogeneous catalysis.

Blechert and co-workers thereafter reported that the trifluoromethylation of (hetero)arenes could also be performed under heterogeneous catalysis [33]. To this aim, the Ru- or Ir-based catalysts were replaced with a mesoporous graphitic carbon nitride polymer (mpg-CN), which offers the advantage of being cheap, metal-free and recyclable. A variety of heteroarenes, like pyrroles, oxazoles, furanes, thiophenes, indoles and pyrazines were successfully converted into the corresponding trifluoromethylated products in moderate to good yields (Scheme 27). Remarkably, a side chlorination reaction was observed during the optimisation phase, which was possible to minimise by increasing the catalyst loading (see later in the text for other chlorinations with CF_3_SO_2_Cl).

Indirectly, CF_3_SO_2_Cl intervened in the trifluoromethylation of (hetero)arenes; indeed, when reacted with zinc in water, it afforded the zinc sulfinate salt (CF_3_SO_2_)_2_Zn, which demonstrated great efficiency in introducing the CF_3_ moiety on (hetero)aromatic rings [34].

**Scheme 28:**
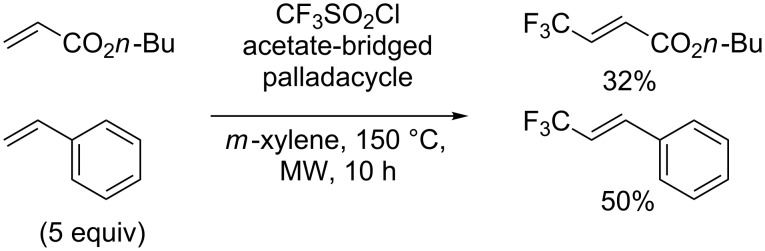
Trifluoromethylation of terminal olefins.

**Trifluoromethylation of olefins:** In 2005, Vogel and co-workers showed that terminal alkenes could be trifluoromethylated by means of CF_3_SO_2_Cl via a palladium-catalysed desulfitative Mizoroki–Heck reaction, in classical solvents or in an ionic liquid media, to yield the corresponding CF_3_ alkenes (Scheme 28) [35–36].

**Scheme 29:**
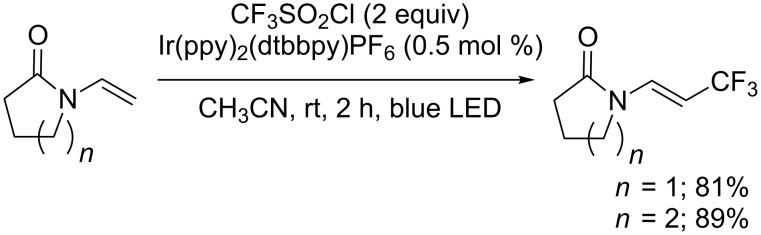
Trifluoromethylation of enamides.

As for Yu, Zhang and co-workers, they described the trifluoromethylation of two enamides under photocatalytic conditions, using similar conditions as those they proposed for the introduction of the CF_3_ moiety on enol acetates (Scheme 29) [37].

**Scheme 30:**
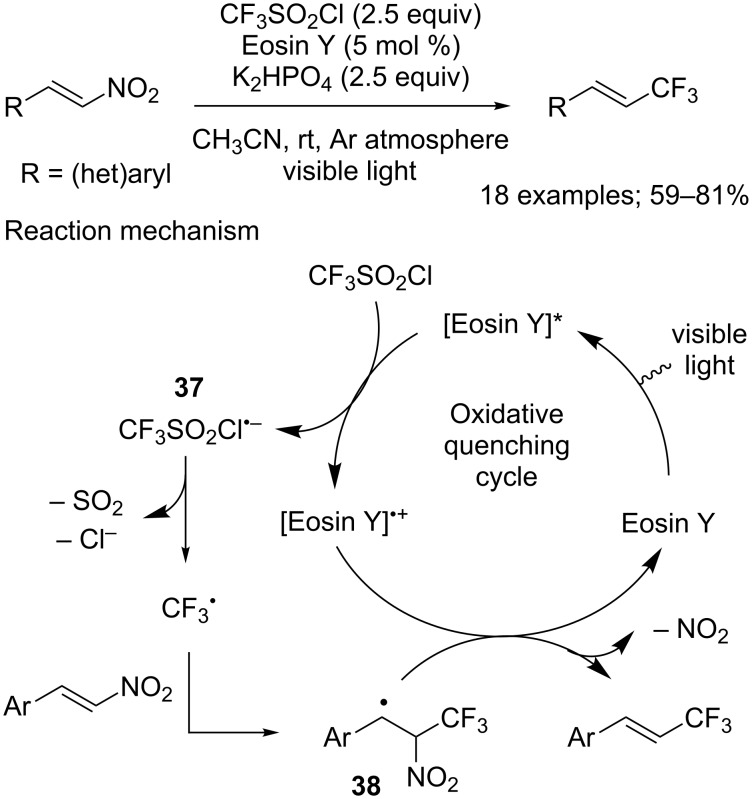
(*E*)-Selective trifluoromethylation of β-nitroalkenes under photoredox catalysis.

Anecdotally, CF_3_SO_2_Cl was evaluated for the trifluoromethylation of allylsilanes, but, disappointingly, gave lower yields than Togni’s hypervalent iodine reagent [38]. More recently, Balaraman and co-workers studied extensively the reaction of β-nitroalkenes with trifluoromethanesulfonyl chloride [39]. They found out that in the presence of the photocatalyst Eosin Y, under visible-light irradiation, such substrates could be selectively converted into (*E*)-1-trifluoromethylalkenes in moderate to good yields (Scheme 30). A plausible mechanism for this reaction was proposed: first, Eosin Y reached its photoexcited singlet state by visible light irradiation, then proceeded to reduce CF_3_SO_2_Cl through SET. As usual, the formed radical anion **37** immediately collapsed to give CF_3_^•^, generating SO_2_ and a chloride anion in the process. The trifluoromethyl radical then reacted with the β-nitroalkene to furnish radical intermediate **38**, which was reduced by the Eosin Y radical cation to yield the expected product after elimination of NO_2_. This proposed mechanism can rationalise several limitations of the reaction, such as its incompatibility with aliphatic β-nitroalkenes because of the lower stability of the radical intermediate **38** generated. The variation of stability of this species also explained the higher yields obtained with β-nitrostyrene derivatives substituted by electron-withdrawing groups.

**Scheme 31:**
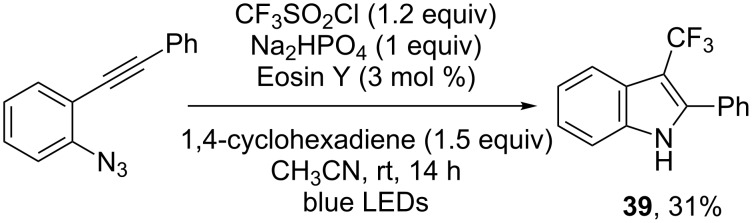
Photoredox-catalysed trifluoromethylation/cyclisation of an *o*-azidoarylalkynes.

**Trifluoromethylation of alkynes:*****o*-Azidoarylalkynes also proved to be interesting substrates for cascade reactions, allowing to obtain the 3-trifluoromethylated indole **39** albeit in low yield (Scheme 31) [40].

**Scheme 32:**
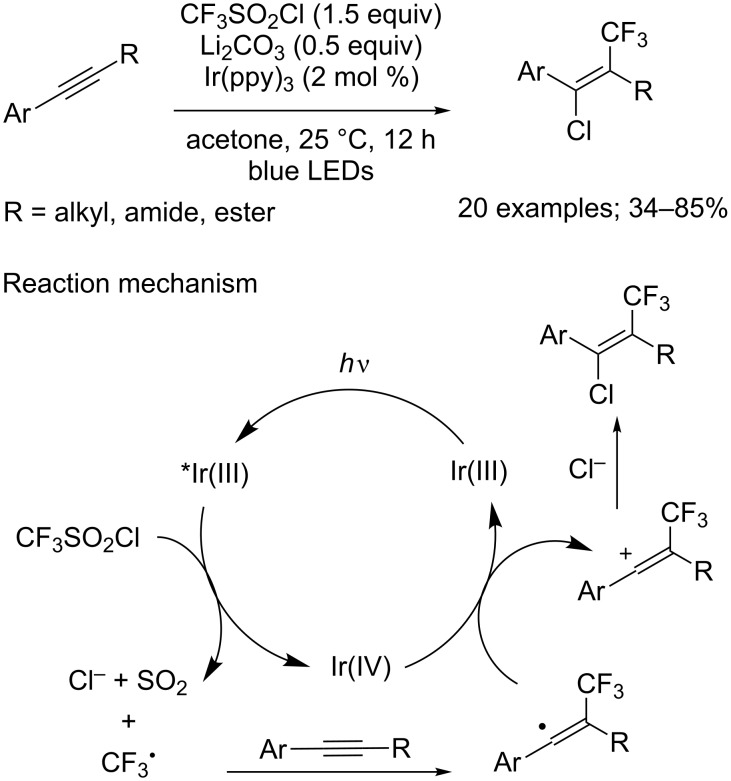
Regio- and stereoselective chlorotrifluoromethylation of alkynes.

**Chlorotrifluoromethylation of alkynes:** In the continuity of their work on alkenes, Jung and Han got interested in the chlorotrifluoromethylation of internal alkynes [41]. In the presence of 2 mol % of Ir(ppy)_3_ and Li_2_CO_3_, under blue LED irradiation, this type of substrate was easily converted into the corresponding alkenes through a mechanism similar to the one described for alkenes (Scheme 32). It is noteworthy that the introduction of the chlorine atom took selectively place from a direction anti to the CF_3_ group, probably because of electrostatic repulsion. The reaction proceeded smoothly with prop-1-yn-1-ylbenzene derivatives, indifferently to the substitution pattern of the aryl moiety. As for the R group, alkyl, ester or amide moieties were well-tolerated. Terminal alkynes could also be submitted to these conditions successfully, albeit in lower yields.

### 2 Trifluoromethylsulfenylation

In 2016, CF_3_SO_2_Cl was proposed for the first time as a new electrophilic trifluoromethylsulfenylation reagent by our research group [42]. To achieve that kind of transformation, said reagent was used under reductive conditions in order to generate in situ the highly reactive CF_3_SCl, which could subsequently be trapped by nucleophiles.

**Scheme 33:**
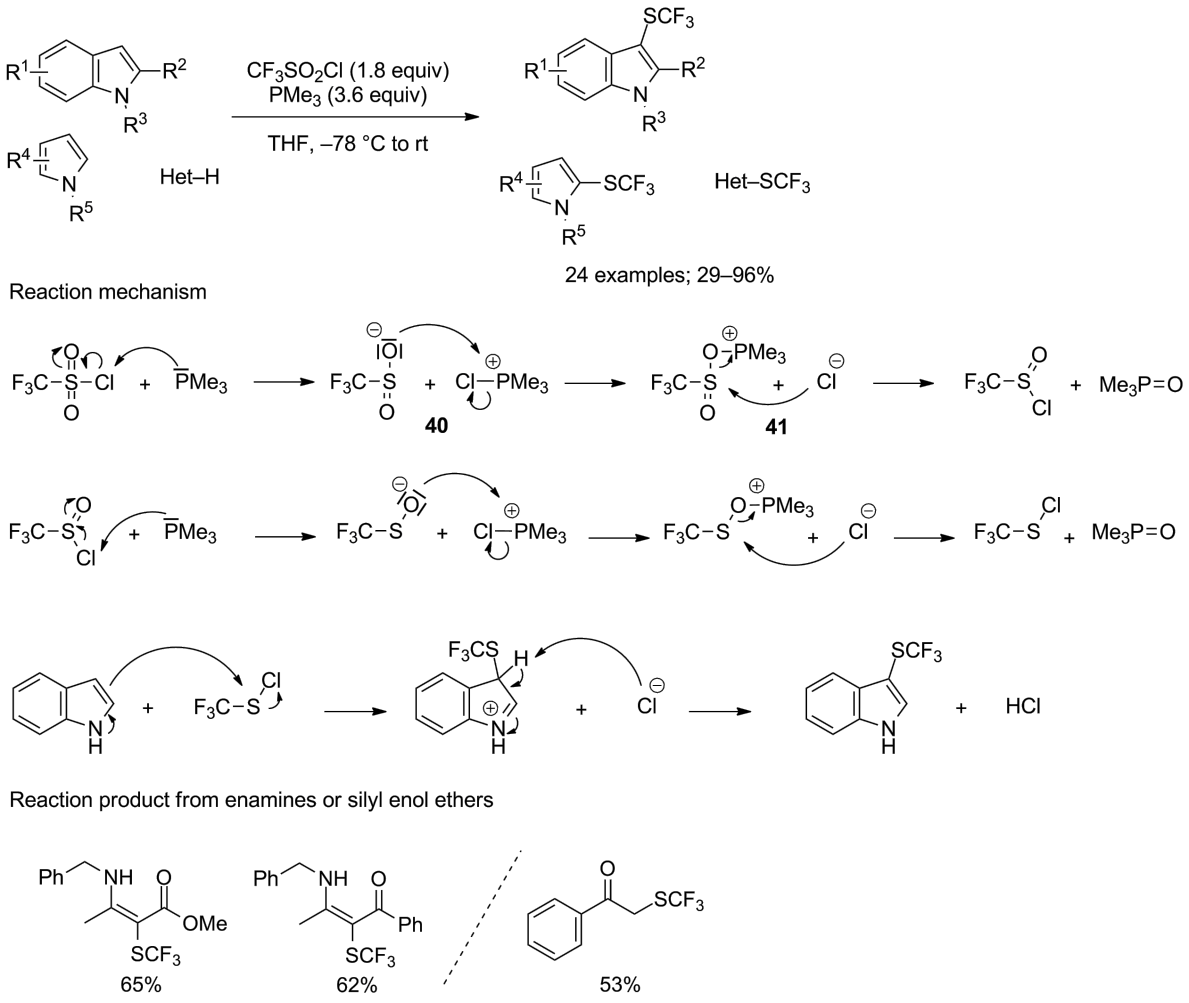
PMe_3_-mediated trifluoromethylsulfenylation by in situ generation of CF_3_SCl.

Indole derivatives proved to be appropriate substrates for this reaction, and a variety of them were selectively converted into their 3-trifluoromethylated analogues in the presence of trimethylphosphine, with moderate to excellent yields (Scheme 33). The higher nucleophilicity of trimethylphosphine versus triphenylphosphine and the water solubility of trimethylphosphine oxide byproducts were essential elements in choosing the reducing agent. Both electron-withdrawing and donating groups were well-tolerated on various positions of the benzo-fused ring, without tremendous influence on the yields. Similarly, substrates featuring alkyl and aryl substituents in position 1 or 2 were compatible with the reaction conditions. On the other hand, 2-trifluoromethylsulfenylation did not occur with 3-substituted substrates. Other azaarenes, such as pyrrole derivatives, as well as enamines or silyl enol ethers were also compatible with these conditions, and furnished the corresponding products in moderate to good yields. The key step of the reaction comprises the formation of an halogen bond between the positive electrostatic potential on the outer side of the chlorine atom and the lone pair of the phosphorus atom of the phosphine. This phenomenon indeed triggered the cleavage of the S–Cl bond, producing chlorophosphonium sulfinate **40**, which was then readily converted into an *O*-sulfinatophosphonium chloride **41**. The latter finally gave access to the corresponding sulfinyl chloride through an Arbuzov collapse. The obtained sulfinyl chloride then underwent a similar sequence to yield CF_3_SCl, which then reacted with the chosen nucleophile to provide the trifluoromethylsulfenylated analogue (Scheme 33).

**Scheme 34:**
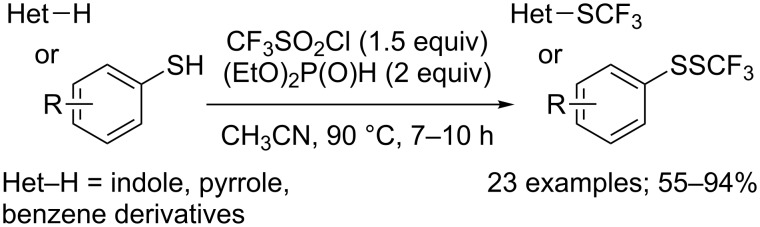
(EtO)_2_P(O)H-mediated trifluoromethylsulfenylation of (hetero)arenes and thiols.

Yi and co-workers reported that the reaction could also be performed in acetonitrile at 90 °C, with diethyl phosphite as the reducing agent (Scheme 34) [43]. These modifications allowed to get improved yields for the trifluoromethylsulfenylation of indole and pyrrole derivatives. Morevover, the scope could be extended to other substrates of interest, such as activated benzene derivatives and thiols.

**Scheme 35:**
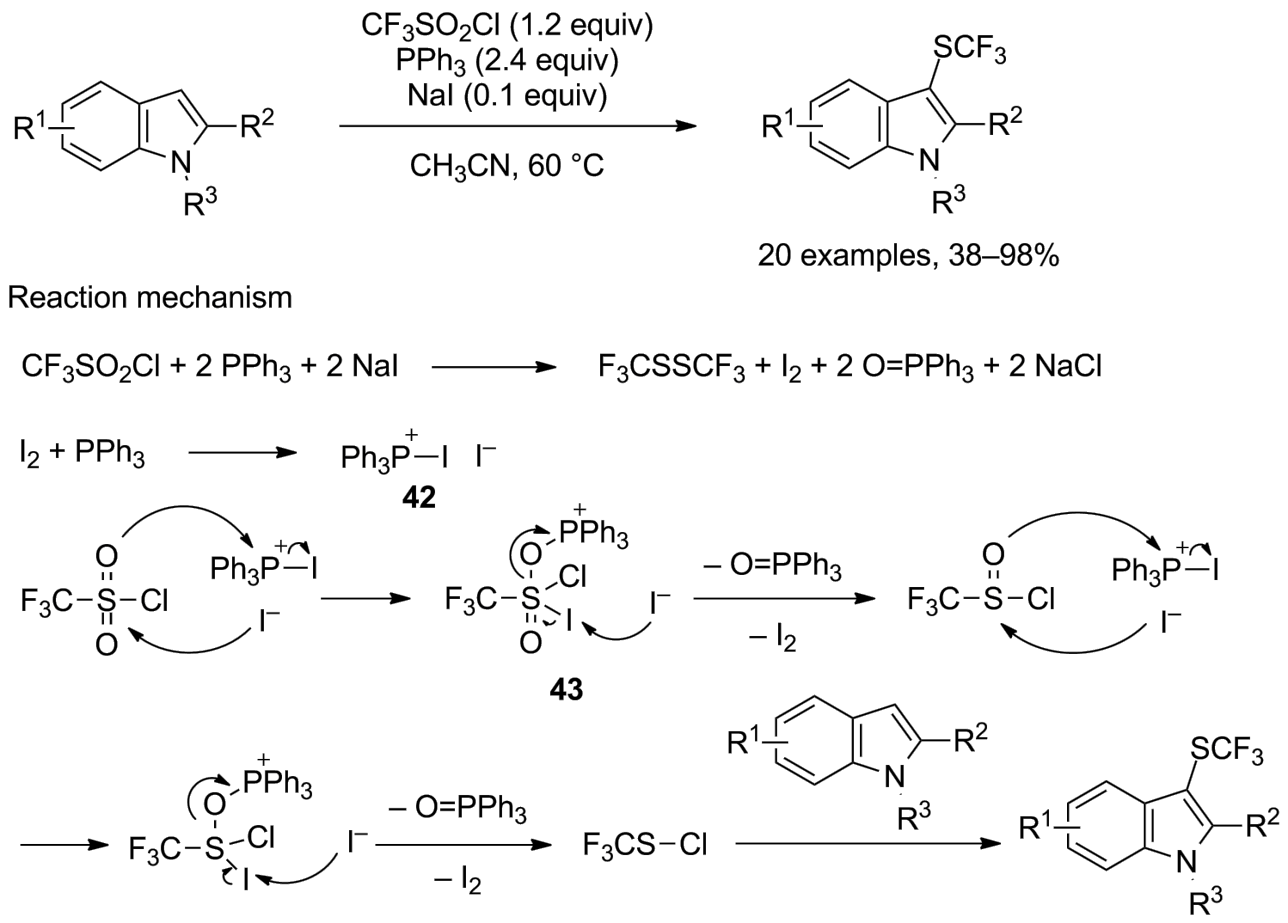
PPh_3_/NaI-mediated trifluoromethylsulfenylation of indole derivatives.

Similarly, Lu, Zhao and co-workers found out that excellent yields could also be achieved for indole derivatives when replacing PMe_3_ or (EtO)_2_P(O)H by cheap and stable triphenylphosphine in acetonitrile at 60 °C [44]. The addition of catalytic amounts of sodium iodide, while not being essential for the production of the trifluoromethylsulfenylated substrates, permitted to slightly increase the yields (Scheme 35). For that matter, excellent yields were achieved indifferently of the nature and position of the substrate substituents. Notably, this procedure allowed for the synthesis of 2-trifluoromethylsulfenylated 3-methylindole, which could not have been realised with the two previously evocated methodologies. The isolated yield was nonetheless quite low (38%). As opposed to previous reports, the proposed mechanism does not include the free phosphine as the reducing agent, but rather iodotriphenylphosphonium iodide **42**. This species was supposedly generated from PPh_3_ and I_2_, itself issued from the reaction of CF_3_SO_2_Cl, PPh_3_ and NaI. Species **42** was able to reduce CF_3_SO_2_Cl through the nucleophilic attack of the sulfur atom by the iodine counter anion, leading to the formation of intermediate **43**, which ultimately furnished CF_3_SOCl, regenerating I_2_ in the process. A second reduction then took place, followed by the electrophilic trifluoromethylsulfenylation step. According to this proposed mechanism, bis(trifluoromethyl)disulfide (CF_3_SSCF_3_) was generated but its possible role in the trifluoromethylsulfenylation was not evoked.

**Scheme 36:**
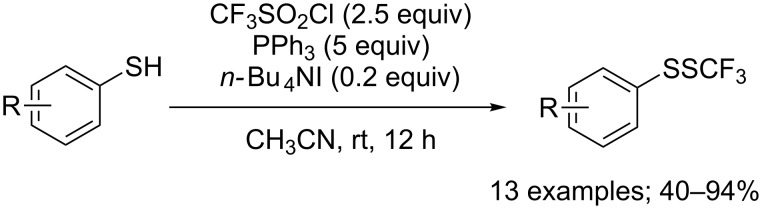
PPh_3_/*n-*Bu_4_NI mediated trifluoromethylsulfenylation of thiophenol derivatives.

A slight tuning of the reaction conditions, including notably a replacement of NaI by *n-*Bu_4_NI, as well as the increase of reagents quantities permitted to perform the trifluoromethylsulfenylation of thiophenol derivatives at room temperature (Scheme 36) [45]. These conditions proved to be tolerant with variously substituted aryl thiols, but no conversion was observed for any aliphatic substrates.

### 3 Trifluoromethylsulfinylation

**Scheme 37:**
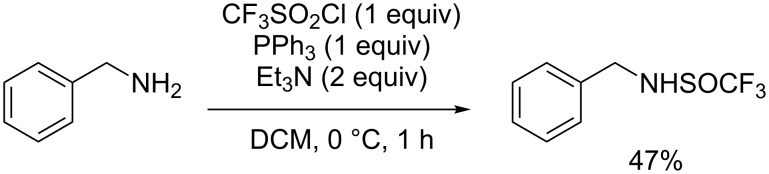
PPh_3_/Et_3_N mediated trifluoromethylsulfinylation of benzylamine.

Following a similar concept, CF_3_SO_2_Cl could also be used in an interrupted reduction to selectively furnish CF_3_SOCl, thus allowing the trifluoromethylsulfinylation of nucleophiles. The first reports on the introduction of the SOCF_3_ group using such strategy dated back to 2007 and 2009, but were, however, limited to benzylamine [46–47]. Using 1 equivalent of CF_3_SO_2_Cl and PPh_3_ in the presence of 2 equivalents of Et_3_N, benzylamine was converted into the corresponding product in 47% yield (Scheme 37).

**Scheme 38:**
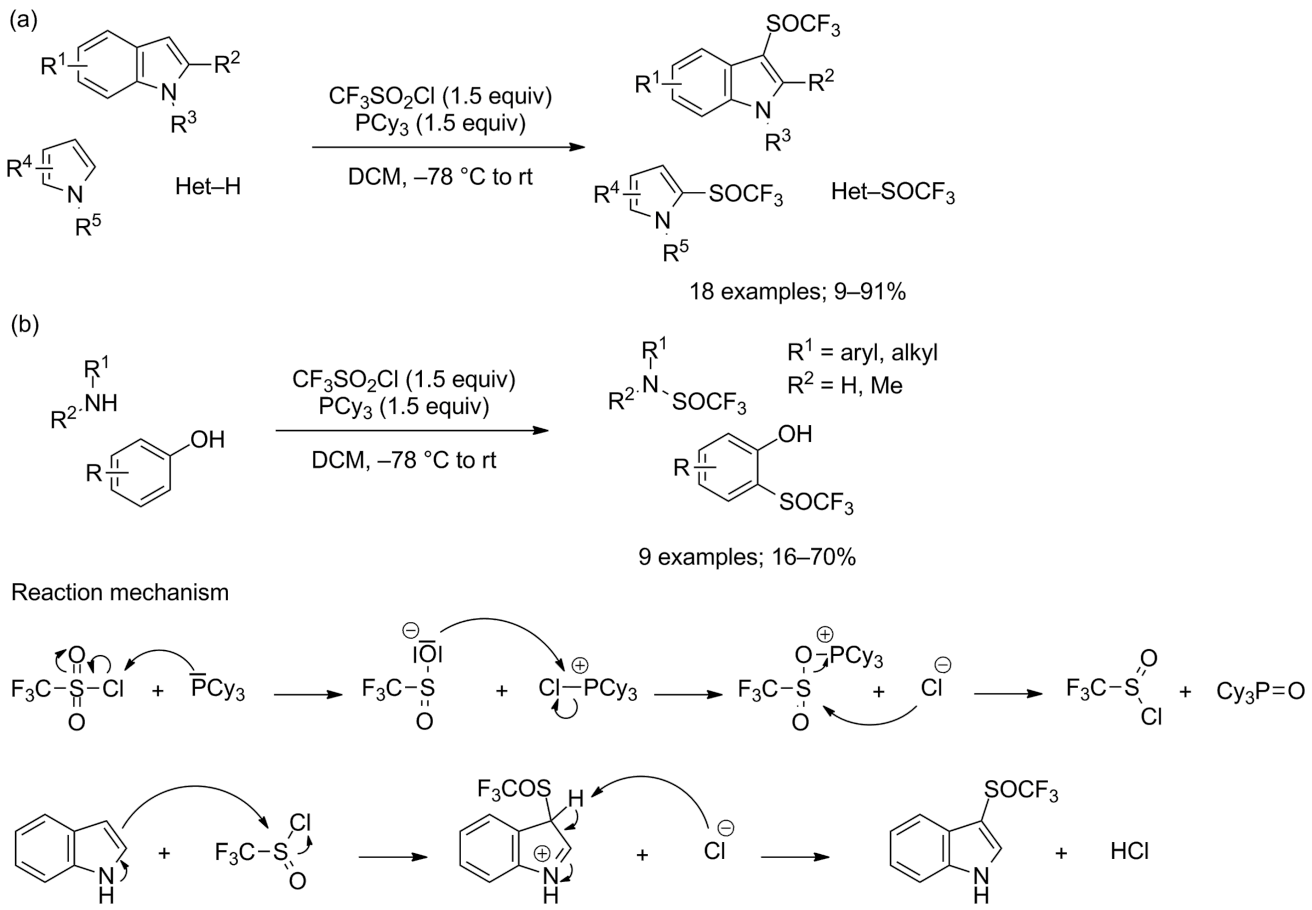
PCy_3_-mediated trifluoromethylsulfinylation of azaarenes, amines and phenols.

A more extensive study of this type of reaction was carried out by our research group in 2017 [48]. Using 1.5 equivalents of CF_3_SO_2_Cl and tricyclohexylphosphine, the trifluoromethylsulfinylation of various indole and pyrrole derivatives featuring diverse functional groups, as well as other azaarenes could be achieved in low to excellent yields (Scheme 38a). Generally, indole derivatives provided better results than pyrrole derivatives, which were often involved in polymerisation and poly functionalisation side reactions. The scope of the reaction could as well be extended to aryl and alkylamines, albeit the products were obtained in reduced yields, which were partly due to the instability of the formed compounds in the reaction medium. As for phenol derivatives, their lower reactivities led to even further decreased yields (Scheme 38b). Interestingly, while the introduction of the SOCF_3_ moiety occurred selectively on the nitrogen atom for amines, only the *C*-trifluoromethylsulfinylated products were isolated when performing the reaction on phenol derivatives. Such products were probably obtained through an *O*-trifluoromethylsulfinylation step, followed by a rearrangement. As for the mechanism of the reaction, we proposed a pathway similar to the one we previously described for the trifluoromethylsulfenylation of indoles, except that the nature of the phosphine as well as the stoichiometry between CF_3_SO_2_Cl and PCy_3_ prevented the reduction of formed CF_3_SOCl and therefore allowed its direct reaction with the substrate (Scheme 38).

### 4 Chlorination

**Scheme 39:**
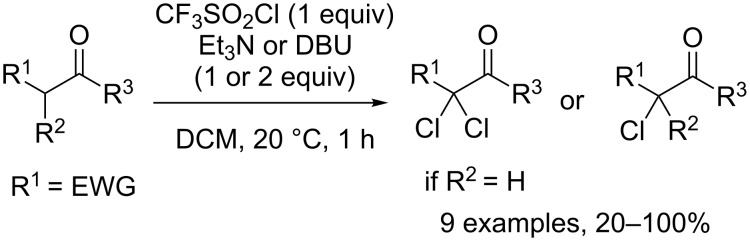
Mono- and dichlorination of carbon acids.

Sparingly, CF_3_SO_2_Cl was employed as a chlorinating agent. The first example of such type of reaction was reported by Just and Hakimelahi in 1979 [49]. Their work was focused on the mono- or dichlorination of various carbon acids, in the p*K*_a_ range between dialkyl malonate and methyl dichloroacetate, as well as certain nucleophiles, were reacted with trifluoromethanesulfonyl chloride in the presence of a base, like Et_3_N or DBU (1,8-diazabicyclo[5.4.0]undec-7-ene) in dichloromethane (Scheme 39). The chlorinated products were recovered in excellent yields. Interestingly, when the reactions were conducted in methanol, the selectivity proved to be quite high, as the rate of chlorination of carbanions was calculated to be more than 10^5^ higher than that of the sulfonylation of methanol.

**Scheme 40:**
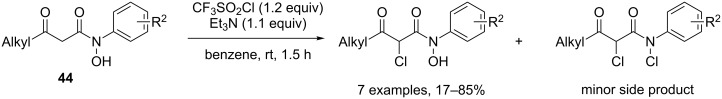
Monochlorination of (*N*-aryl-*N*-hydroxy)acylacetamides.

However, forty years later, Shainyan and Danilevich reported that the process might not be that selective in regard to mono- versus dichlorination of compounds carrying two acidic protons [50]. Indeed, depending on the nature of the substrate, the introduction of one or two chlorine atoms occurred predominantly when performing the reaction with only 1.0 equivalent of CF_3_SO_2_Cl and Et_3_N. Nonetheless, this transformation was utilised for the dichlorination of a cyclopentadiene-1-carbaldehyde derivative [51] and the monochlorination of (*N*-aryl-*N*-hydroxy)acylacetamides **44** (Scheme 40) [52]. In this case, some side *N*-chlorination was observed for certain substrates.

**Scheme 41:**
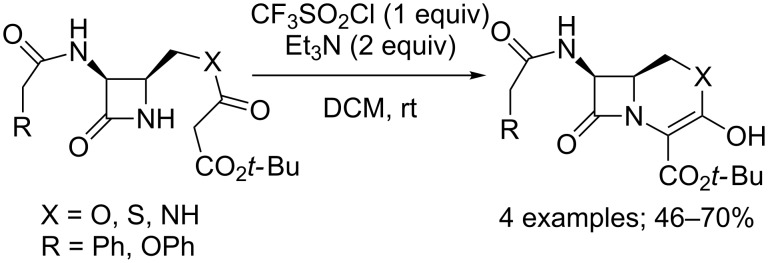
Examples of the synthesis of heterocycles fused with β-lactams through a chlorination/cyclisation process.

Anecdotally, CF_3_SO_2_Cl could also be involved in the chlorination of *ortho*-lithiated veratrole [53]. It also allowed the surprising formation of a 5’-chloro nucleoside, when used in an attempt to prepare the corresponding 5’-OTf nucleoside [54]. Moreover, considering the excellent selectivity of the combination CF_3_SO_2_Cl/Et_3_N towards the chlorination of substrates displaying a hydroxy group, the reaction could be further exploited for cascade chlorination/cyclisation processes. For instance, diethyl malonates substituted by an alkyl chain bearing an alcohol or ether function could give access to tetrahydropyran or -furan derivatives [55]. Furthermore, this type of process also allowed the synthesis of diverse heterocycles fused with β-lactams (Scheme 41) [56]. The competition between mono- and dichlorination remained an issue in these transformations; but fortunately, increasing the bulkiness of the ester group permitted to limit the reaction to the introduction of only one chlorine atom. It was also possible to achieve the isolation of similar compounds starting from differently substituted β-lactams, notably carrying a malonate moiety linked to the nitrogen [57].

**Scheme 42:**
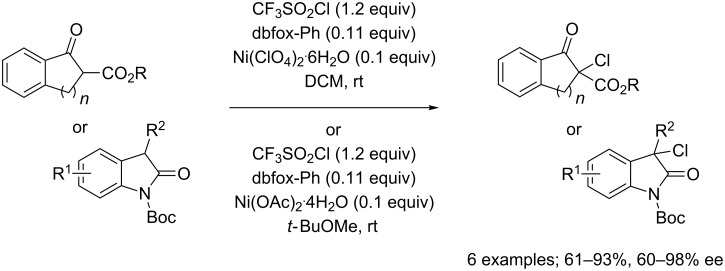
Enantioselective chlorination of β-ketoesters and oxindoles.

More recently, CF_3_SO_2_Cl also found to be an appropriate reagent for the asymmetric introduction of a chlorine atom onto several substrates. For instance, Shibata, Toru and co-workers used CF_3_SO_2_Cl for the enantioselective chlorination of β-ketoesters and oxindoles in the presence of a dbfox-Ph/Ni(II) system (dbfox-Ph = [(*R*,*R*)-4,6-dibenzofurandiyl-2,2'-bis(4-phenyloxazoline)]) (Scheme 42) [58]. The reaction proceeded in good to excellent yields and enantioselectivities.

**Scheme 43:**
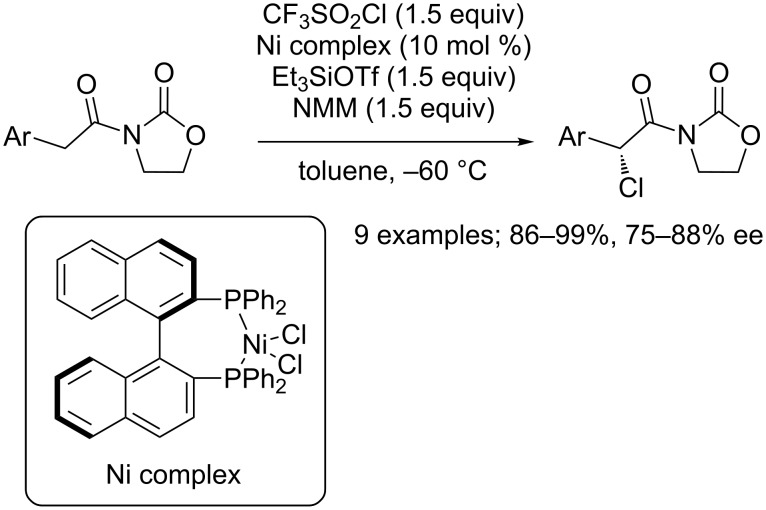
Enantioselective chlorination of 3-acyloxazolidin-2-one derivatives (NMM = *N*-methylmorpholine).

In 2011, Sodeoka and co-workers reported that this reagent was also suitable for the asymmetric chlorination of 3-acyloxazolidin-2-one derivatives thanks to a trinary activation system (Scheme 43) [59]. The expected products were isolated in high yields and enantioselectivities, and no dichlorination reaction occurred. Interestingly, in both cases, it was observed that far lower ee values were reached when replacing CF_3_SO_2_Cl by *N*-chlorosuccinimide, which highlighted its great compatibility with asymmetric reactions.

## Conclusion

Trifluoromethanesulfonyl chloride is an inexpensive versatile reagent indissociable from major achievements in the field of trifluoromethylation. Indeed, early discoveries by Kamigata in the nineties using ruthenium catalysis and, more recently in 2011, by MacMillan using photoredox catalysis for the direct trifluoromethylation of the inherently reactive positions of the substrates, paved the way to a dramatic acceleration of discoveries in the field. In addition, the recent breakthrough methods for direct trifluoromethylsufenylation and trifluoromethylsufinylation offer alternative accesses to SCF_3_ and S(O)CF_3_ compounds, respectively, bypassing the use of sophisticated SCF_3_ donor reagents. Lastly, CF_3_SO_2_Cl has a demonstrated ability to transfer an electrophilic chlorine atom for efficient chlorination reactions including enantioselective chlorination. Current know-how and further exploration of the utility of this reagent will undoubtedly be beneficial for the pharmaceutical and agrochemical industries in which new opportunities for economical and sustainable development are eagerly sought after. Numerous applications and novel reactions are expected to appear, thus contributing to enrich the bright future of trifluoromethanesulfonyl chloride.
